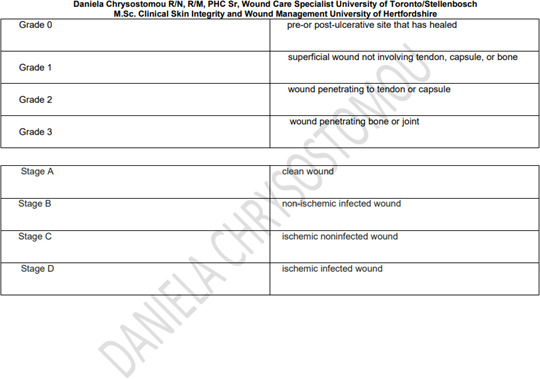# Medical-Grade Honey Is a Versatile Wound Care Product for the Elderly

**DOI:** 10.14283/jarlife.2024.7

**Published:** 2024-05-17

**Authors:** D. Chrysostomou, A. Pokorná, N.A.J. Cremers, L.J.F. Peters

**Affiliations:** 1 Wound Clinic Health@45, Linksfield Road 45, Dowerglen, Johannesburg 1612, South Africa; 2 Department of Health Sciences, Faculty of Medicine, Masaryk University, Brno, Czech Republic; 3 Department of Public Health, Faculty of Medicine, Masaryk University, Brno, Czech Republic; 4 College of Polytechnics Jihlava, Jihlava, Czech Republic; 5 Triticum Exploitatie BV, Sleperweg 44, 6222NK Maastricht, The Netherlands; 6 Department of Gynecology and Obstetrics, Maastricht University Medical Center, 6202 AZ Maastricht, The Netherlands.

**Keywords:** Wound care, medical-grade honey, geriatric patients, chronic wounds, acute wounds

## Abstract

**Introduction:**

Ageing of the global population has led to an increase in the demand for the treatment of wounds, especially considering the challenges of managing wounds in the elderly. Therefore, more effective treatment strategies need to be explored. In this article, we aimed to compare medical-grade honey (MGH) products with other wound care products and to provide guidelines on using MGH in wounds commonly found in the elderly.

**Methods:**

Based on literature research and expert opinion, an overview of commonly used wound care products and their wound healing characteristics is provided. In addition, literature-based classification of wounds in the elderly and the recommendations for treatments are provided.

**Results:**

Frequently used wound care products include povidone-iodine, enzymatic products, absorbing dressings, larvae, silver dressings, and MGH dressings. Supported by systematic reviews and meta-analyses, MGH dressings were identified as the most potent and all-round wound care product compared to the others. Next, we provided basic guidelines for managing the most common wounds in the elderly, both acute and chronic, and specified how and which MGH products can be used in these wounds.

**Conclusion:**

MGH is a widely applicable, safe, easy-to-use, and cost-effective product to manage wounds in the elderly. In case of doubt, refer to a trained wound care specialist who can support the treatment of difficult-to-heal wounds.

## Background

**T**he standard of wound care has increased tremendously in the last few years. Scientific research combined with expert opinion led to the creation of guidelines for wound management. All medical practitioners will come across wounds in their daily practice, especially regarding elderly patients. Looking ahead to the year 2050, the population of older adults will rise from 1 billion to 2.1 billion (1). Population ageing has major consequences for healthcare, including rising demand, economic effects, and personal expenditures (2). Moreover, elderly will encounter a variety of health issues, including chronic wounds. These wounds fail to heal in a predictable timeframe (4 weeks to 3 months) or in a typical set of stages without responding to standard therapies (3).

Chronic wounds, also known as hard-to-heal or non-healing wounds, are associated with considerable morbidity and mortality in elderly (4). These wounds are common among elderly due to various factors such as comorbidities, e.g. vascular insufficiency or diabetes (4, 5). Chronic wounds are characterized by a disrupted healing process, which normally exists in four overlapping phases (6, 7): hemostasis, inflammation, proliferation, and maturation. Wounds turn chronic often due to bacterial infections, but also comorbidities and/or higher fragility and slower healing of the skin consequent to aging (7-9). Thus, as the elderly population continues to grow, the burden of managing wounds becomes even more critical. Therefore, exploring effective strategies to improve wound healing and prevent acute wounds from becoming chronic in older adults is crucial. Special attention must be paid to the effective management of infection as it is known historically that in the elderly, the symptomatology of infection is altered in their clinical presentation due to age-related alterations in immunology (10).

Honey has been used to treat wounds throughout history but became forgotten as a wound care product because of the discovery of antibiotics (11-14). With the advent of antibiotic-resistant bacteria, there has been increasing interest in honey as a wound care product (14-18). The wound care properties of honey are based on two main aspects: its antimicrobial and pro-healing effects (15, 19, 20). Because honey targets the bacteria in a multifaceted manner, it works against a broad spectrum of bacteria and lacks the risk of resistance. Commercially available honey has lower antibacterial activities due to production processes and adulteration (21-23). Therefore, medical-grade honey (MGH) is preferred for wound care as it meets strict safety criteria, such as being organic and free from pollutants, and is sterilized to ensure safety and efficacy for medical use (21-24). While MGH has shown effectiveness in elderly patients, non-honey products, such as povidone iodine or silver, are still commonly used. The broad range of wound care products available can overwhelm the health care professional and exact guidelines for treating wounds in elderly are hard to find.

This article aims to provide an overview and comparison between non-honey and MGH-based wound care products that can be used in the elderly. Furthermore, we will focus on the different types of wounds encountered in elderly and suggest management strategies. We will also demonstrate how MGH can be used in every wound care situation in elderly.

## Wound care products

Wounds can be treated with a variety of products and the choice can be challenging for the healthcare professional. To choose an appropriate dressing, we first need to know what the ideal dressing would be. The characteristics of an ideal wound care product include (25-27):

- Can absorb and control exudate
- Cost-effective
- Can be removed without causing damage to the wound
- Easy to use
- Reduces and controls bacterial load
- Removes sloughy and necrotic tissue
- Exhibits anti-inflammatory properties
- Eliminates unpleasant odor from the wound
- Non-toxic and promotes the growth of new tissue

The most commonly used products will be discussed and compared to the ideal wound care dressing characteristics (Table 1).

**Table 1.** Wound care products and their effects on the wound healing process

	Moist wound	environment	Antibacterial	Debridement	Anti-inflammatory	Removes malodor	Stimulates tissue growth
Povidone-iodine			V			V	X
Enzymatic products		V		V			
Absorbing dressings		V		V			
Larvae		V		V	V	V	
Silver dressing/SSD		V	V		V	V	X
MGH		V	V	V	V	V	V

Results are based on the IFUs of the products. V marks a positive effect, while X marks a negative effect on the described characteristic. SSD = silver sulphadiazine; MGH = medical-grade honey.

### Povidone-iodine

Povidone-iodine is a common antiseptic agent used in wound care and has a broad-spectrum antibacterial activity. It releases free iodine, which quickly penetrates microorganisms and eventually causes cell death (28, 29). Povidone-iodine is available in various forms and is often used for wound cleansing and preoperative skin preparation. One consideration is that it is damaging to healthy tissue, thus slowing wound healing (30).

### Enzymatic products

Enzymatic products are designed to debride the wound. Debridement means removing necrotic material, devitalized tissues, scabs, and other impurities that delay wound healing (31). These products contain specific enzymes, such as collagenases or proteases, that digest and degrade devitalized tissue. Some enzymes selectively target non-viable tissue, while others also target viable tissue (31). One should note though that enzymatic products lack antimicrobial properties (32).

### Absorbing dressings

Absorbing dressings are designed to manage wound exudate and maintain a moist wound environment, which is considered key in wound management (33, 34). These dressings are composed of highly absorbent materials, such as foam or alginate, which effectively absorb and retain excess fluid from the wound bed (34). By minimizing excessive moisture, absorbing dressings help prevent maceration of the surrounding skin and promotes optimal conditions for wound healing. Absorbing dressings are useful in creating and maintaining a moist wound environment. These dressings can be used as complementary dressings (34).

### Larvae

Larval therapy, also known as maggot debridement therapy, involves the controlled application of medical-grade fly larvae to wounds (35). The larvae secrete enzymes that break down necrotic tissue, effectively debriding the wound. Moreover, maggot therapy has been shown to have anti-inflammatory properties as well (35, 36). Larval therapy is particularly beneficial for chronic, non-healing wounds with significant necrosis. One of the limitations of this treatment is that maggots can induce dermatitis when not properly secured (37). Also, not many patients are comfortable with larval therapy.

### Silver dressings

Silver dressings are dressings that contain silver compounds or nanoparticles and have broad-spectrum antimicrobial properties. These dressings provide a sustained release of silver ions, which exert their antimicrobial effects by disrupting microbial cell membranes and interfering with essential cellular processes (38). Silver dressings are frequently used in infected wounds to reduce the bacterial burden and promote wound healing. However, silver dressings are advised to be used for no longer than two weeks, after which treatment should be switched to a non-silver dressing (39). The use of silver can delay wound healing, lead to skin irritation, and carries a high risk of developing argyria.

### MGH-based dressings

MGH-based dressings are divided into two main categories: Manuka honey and other kinds of honey. The main difference between the two is that Manuka’s antibacterial activity relies mostly on methylglyoxal while other honeys rely on hydrogen peroxide production (40). MGH-based dressings harness the natural properties of honey, including its antimicrobial effects and promotion of wound healing (19, 20). Additionally, these dressings have been shown to effectively manage infected wounds, promote autolytic debridement, stimulate a moist wound environment, and enhance re-epithelialization (41-43). The unique composition of MGH-based dressings contributes to their versatility and therapeutic efficacy in various wound types and stages of healing. One should note that pure MGH or MGH in high concentrations can cause a stingy feeling upon application.

Although each product ticks multiple boxes, only MGH matches all characteristics of the ideal wound care dressing. Systematic reviews and meta-analyses have concluded that MGH has antimicrobial properties, stimulates wound healing, has several benefits over the use of other wound care products, can be used for a wide range of acute and chronic wounds, and is cost-effective (44-50). Besides this, studies have compared MGH to silver products and povidone iodine and concluded that MGH was superior as a wound care dressing (44, 50).

## Wound care protocol

Patient characteristics influence the healing trajectory. Therefore, management of wounds in elderly patients must start with a full history, physical examination, and identification of systemic, psychological, lifestyle, and local factors. Appendix 1 shows an assessment form to be able to pinpoint all the unique patient-related issues. Based on this information, a proper plan of care is developed. Consideration should also be given to means of transportation to a wound care facility and the patient’s capability to participate in the management of their wound(s). Social support in all forms will be paramount in the completion of a multifaceted plan of wound care.

**Figure 1.**
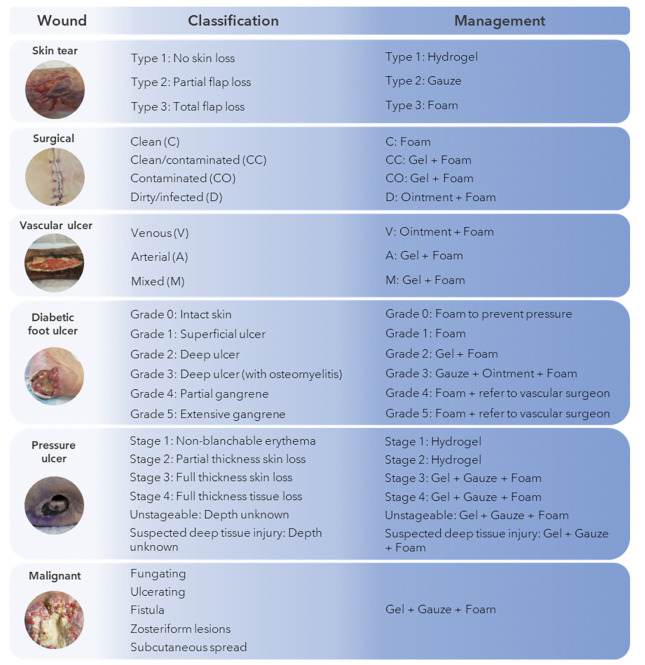
Medical-grade honey for use in elderly

Wounds can be categorized into distinct classifications, namely acute and chronic wounds, each encompassing a diverse array of types. We will provide an overview of the different types of wounds that regularly occur in elderly and how to manage them according to wound type and classification. Since MGH-based wound care products have the characteristics of the ideal wound care dressing, we will highlight in Figure 1 which MGH-based product can be applied in which wound care situation exactly. Numerous studies have shown that supplementation of MGH with other compounds, such as vitamins C and E, leads to heightened antimicrobial and wound healing activities of the MGH compared to its non-supplemented counterpart (51-54). Therefore, we demonstrated the use of supplemented MGH products (L-Mesitran, manufactured by Theo Manufacturing, Maastricht, the Netherlands) which include ointment, gel, tulle, hydrogel, and foam dressings.

Suggested treatment options using supplemented MGH products for wounds that are commonly found in elderly. Each wound is classified (55-59) and based on the classification an appropriate product of the supplemented MGH range is suggested. Hydro = L-Mesitran Hydro; Gauze = L-Mesitran Tulle; Foam = L-Mesitran Foam; Gel = L-Mesitran Soft; Ointment = L-Mesitran Ointment.

### Common acute wounds in the ageing population

#### Skin tear

**Figure 2.**
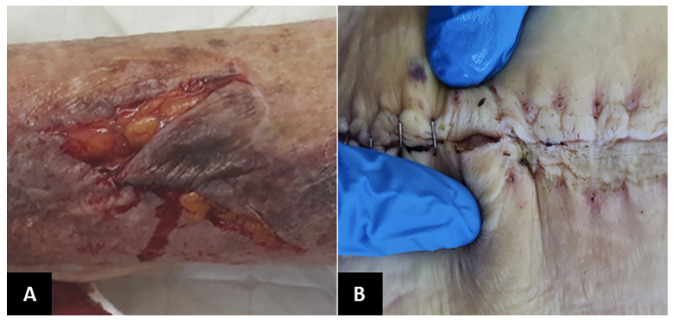
Common acute wounds in elderly

One of the most frequent acute wounds is skin tears (Figure 2A). These traumatic wounds can result from friction, shear, or blunt trauma. Skin tears can happen on any area of the body and are more likely to occur in individuals with delicate skin, in particular the elderly (60). Skin tears can involve the separation of the epidermis from the dermis (partial thickness wounds) or the separation of both the epidermis and dermis from underlying structures (full-thickness wounds) (60).

A) Skin tear with partial flap loss, which cannot be repositioned to cover the wound bed. B) Infected post-operative abdominal wound.

There is one international classification tool validated and recommended to use in the management of skin tears: The International Skin Tear Advisory Panel (ISTAP) (55). It recommends the following steps in managing lacerations:

1. Assess and classify the wound using a reliable tool.
2. Preserve as much as possible of the skin flap (gently with a moistened swab).
3. Aline the edges of the wound and secure it with gentle, adhesive, sterile tape.
4. Protect the wound from further injury using an antimicrobial, sterile dressing.
5. Follow up to ensure adequate wound healing.

The use of MGH for treating skin tears has been documented previously (61, 62). In both publications, skin tears in elderly patients were successfully treated with MGH. The advantage for skin tears are especially the non-adherent properties of MGH-based products. This allows for easy removal of the dressing while not causing any trauma to the wound or surrounding skin. Depending on the stage of the wound, one could opt for an MGH-based hydrogel, gauze, or foam dressing (Figure 1).

#### Surgical site management

Increased age is an operative risk factor. An American government study shows that even though people >65 years represent 13% of the country’s population, 20% of the total surgical procedures are allocated to this group (63). Concerning wound care, the most frequent complication of the surgical site is infection (Figure 2B). Such misfortune will result in suffering, prolonged hospital stays, increased cost of care, and increased use of resources (64).

An array of studies regarding the best choice of postoperative dressing concluded that recognition of the surgical wound classification should enable the clinician to choose the adequate wound cover. Surgical wounds are classified for the risk for a surgical site infection (SSI) (56), as clean, clean/contaminated, contaminated, or dirty. Surgical wounds should be kept clean, change of dressing should be done using a sterile technique. One should follow the following management steps:

1. Assess and classify the contamination risk.
2. Clean the wound with an antiseptic fluid.
3. Use a topical antimicrobial, such as MGH.
4. Cover the wound with a sterile dressing.
5. Check and change dressing according to the exudate level and bacterial load.

Several publications have highlighted the effective use of supplemented MGH-based dressings for surgical wounds (41, 42, 65-69). Although most publications include infected, dehisced surgical wounds, MGH can also be used to reduce the infection rate and improve healing as shown by various clinical studies (69-71). Based on the SSI classification, one could use an MGH-based foam dressing alone or combined with an MGH-based wound gel or ointment (Figure 1).

### Common chronic wounds in older adult

#### Vascular leg ulcers

**Figure 3.**
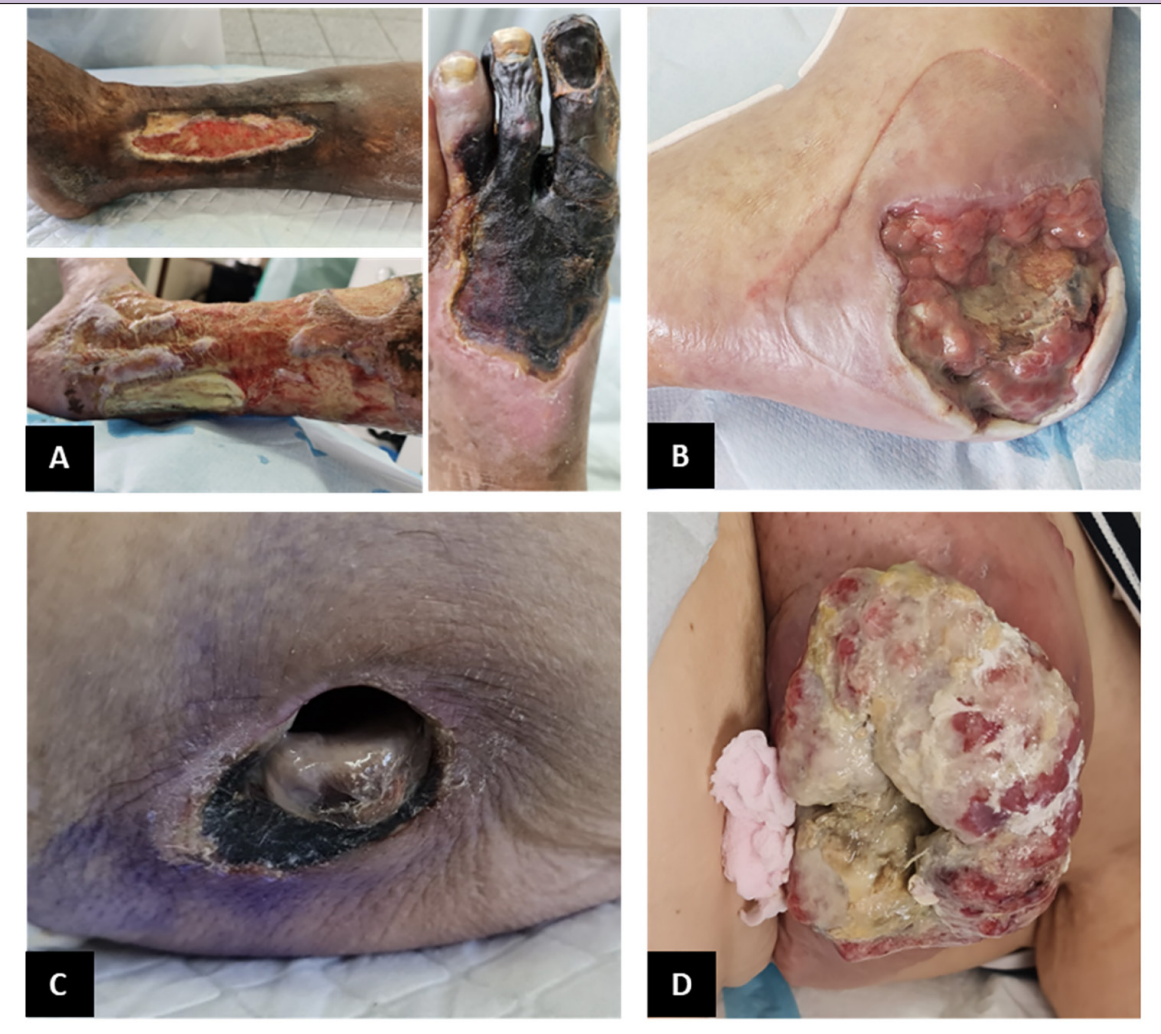
Common acute wounds in elderly

The most frequent vascular ulcers of the lower limb are venous, arterial, or mixed etiology (Figure 3A). Identification and diagnosis are imperiously necessary to be able to deliver appropriate treatment. Venous ulcers are mainly situated above the malleoli, presenting with irregular edges, while arterial ulcers are found over the bony prominence, with round edges and are smaller (72, 73). Mixed ulcers can be present anywhere from on the lower leg, below the knee, all the way to the foot. Venous ulcers are more painful with non-elevation of the leg, while arterial ones are painful on elevation (72, 73).

A) Venous leg ulcer (top), arterial insufficiency (right), and mixed vascular ulcer with tendon exposure (bottom). B) Diabetic foot ulcer with bone exposure. C) Stage 4 pressure ulcer. D) Malignant wound on the breast.

Doppler assessment and ankle brachial pressure index (ABPI) should be performed to establish treatment steps. If ABPI is between 0.8 and 1.1, compression can be used. However, caution is strongly recommended in diabetic patients. If ABPI value is less than 0.8 referral to a vascular surgeon is firmly indicated (72, 73). Furthermore, when lower leg ulcers are painful and have a high bacterial load, wound treatment should consider nonadhesive and antibacterial dressings (72, 73).

The golden standard for treatment according to the etiology of the wound:

1. Venous ulcers - compression and wound care
2. Arterial ulcers - revascularization and wound care
3. Mixed ulcers - revascularization, modified compression, and wound care

Supplemented MGH-based products have been used to treat vascular leg ulcers successfully in the clinic (41, 42, 62, 74-76). These reports showed that even vascular ulcers infected with biofilms or resistant bacterial strains could be healed with supplemented MGH. Moreover, MGH products can be combined with compression thereby making them suitable for treatment of venous leg ulcers. For vascular ulcers, treatment should involve a combination of an MGH-based ointment or gel with an MGH-based foam dressing (Figure 1).

#### Diabetic foot ulcers

Presently, millions of people with diabetes suffer from poorly healing foot ulcers (Figure 3B) (77). The management of diabetic foot ulcers (DFU) is arguably the costliest (78). Unfortunately, it is not limited to the financial aspect alone, the cost is measured in loss of quality of life, loss of limb, and loss of life itself (79).

The first step in the treatment of DFUs is classifying the wound. DFUs are classified from grade 0 to grade 5 according to Wagner’s classification tool (57). A DFU is one of the most challenging and complex wounds, due to numerous intrinsic and extrinsic factors influencing the management outcome. Therefore the following steps should be taken:

- Discuss realistic goals with the patient and carers;
- Plan the treatment;
- Document every step with photographic evidence, accurate wound measurements, and well-written notes.

Early referral to a multidisciplinary team - including a diabetologist, orthopedic surgeon, diabetes nurse, podiatrist, and an orthotist, all working close together with the vascular surgery and infectious diseases departments - has been recommended since 1995 as the best management option (80); wound care by a specialist is an absolute must. The golden standard treatment of DFU is to address three key elements that have a negative impact on healing (80, 81):

1. Vascularization (improving).
2. Pressure (relieving).
3. Infection (control and management).

Two systematic reviews and meta-analyses have demonstrated that MGH shortened wound healing time and increased debridement and bacterial clearance compared to other dressings in DFUs (47, 82). Furthermore, it is safe to use in diabetic patients as it does not increase blood glycemia levels following treatment of DFUs with MGH (83). In all grades of DFUs, apply an MGH-based foam dressing, which may be combined with an MGH-based ointment, gel, or gauze (Figure 1).

#### Pressure ulcers

A pressure ulcer is described in literature as a wound over a bony prominence due to prolonged pressure and many other factors, such as shear or friction (Figure 3C) (58). Wound care has to take into consideration the classification of the pressure ulcer and the treatment plan should be adequately designed. Pressure ulcers are classified into 6 categories according to the National Pressure Ulcer Advisory Panel (NPUAP), the European Pressure Ulcer Advisory Panel (EPUAP), and the Pan Pacific Pressure Injury Alliance (PPPIA) (58). The use of the Braden risk assessment scale in any setting is an excellent guide in the management of pressure ulcers (84). For intensive care unit patients, the Jackson-Cubbin scale is rather used as it has shown superiority to the Braden scale in these specific patients (85). Management of a pressure ulcer includes (58):

1. Identify and address all intrinsic and extrinsic factors by doing a holistic assessment (head-to-toe assessment).
2. Wound assessment, classification, measuring.
3. Perform necessary diagnostic tests.
4. Identify objectives and plan treatment.
5. Document everything.

In various clinical trials, MGH was shown to significantly speed wound healing of pressure ulcers while also providing faster pain relief and less discomfort during dressing changes (86-88). Earlier stages of pressure ulcers should be treated with an MGH-based hydrogel, while later stages require a combination of an MGH-based gel, gauze, and foam dressing (Figure 1).

#### Malignant wounds

Malignant wounds, also known as fungating wounds, are caused when cancerous cells infiltrate and erode through the skin (Figure 3D) (89). These types of wounds are bound to be maintenance wounds (89). The challenges of such wounds are multiple, with the main ones being to control pain, bleeding, odor, and infection. Patient comfort and quality of life are the major priorities in managing the treatment of malignant wounds (89). The Malignant Wound Assessment Tool – Clinical (MWAT-C) can be used for classifying the wound (59). Treatment of such wounds should comprise absorbent, strictly nonadhesive dressings to avoid bleeding, reduce pain at the change of dressing, and control exudate (89). Odor and infection control will contribute to increased quality of life.

The use of MGH in fungating wounds is mainly for its swift deodorizing and cleansing effects (61, 90-92). This in combination with its ability to balance wound moisture levels increases the patient’s quality of life. For malignant wounds one should ideally combine several MGH-based products, i.e. the wound gel, non-adherent gauze, and foam dressing, to obtain fastest results while controlling exudate (Figure 1).

## Conclusion

This article aimed to give healthcare specialists in geriatrics an overview and comparison of wound care products for the elderly, including both non-MGH and MGH-based options. We showed that common therapies do not have all desired properties. For example, although silver-based dressings are excellent antibacterial products, they also damage healthy tissue. MGH, on the other hand, does contain all the desired characteristics for a wound care product. We also demonstrated that MGH can be used in each type of wound commonly formatted in elderly patients.

Wounds in older adults can be challenging and costly similar to other age groups. However, the involution processes and impaired health complexity in older adults often require more extensive personnel and financial resources. The main difficulty with chronic wounds is that they remain trapped in the inflammatory phase of wound healing (6, 7). Usually, the underlying cause is bacterial load, necrotic tissue, presence of biofilm, moisture balance, mechanical issues, or a combination of the above. Still, in older adults, there are other involution-induced problems (hyperemia, hypoxemia etc.). In an ideal scenario, the desired outcome for all wounds is complete closure. However, the complex interplay of various factors determines whether a wound is capable of healing or if it will remain a maintenance wound. It is crucial to consider these factors when approaching wound management to maintain realistic expectations throughout the process. Cause correction facilitates the expected outcome. If in doubt, refer to a trained wound specialist, who will have the ability to assess and manage difficult-to-heal wounds.

## Appendix 1



**Figure:**
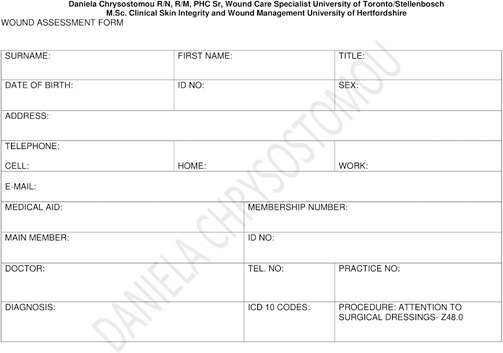


**Figure:**
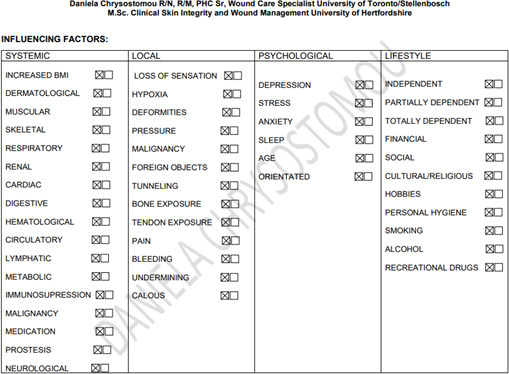


**Figure:**
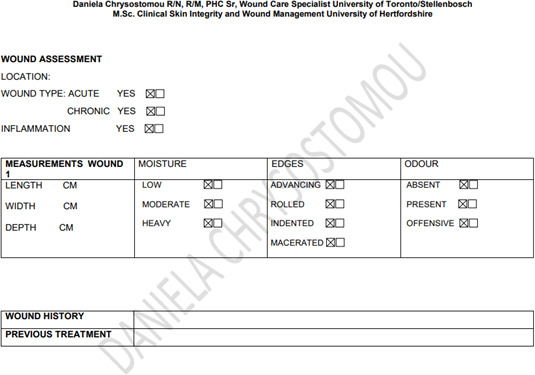


**Figure:**
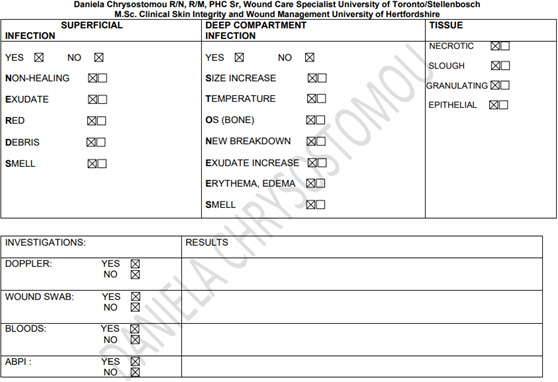


**Figure:**
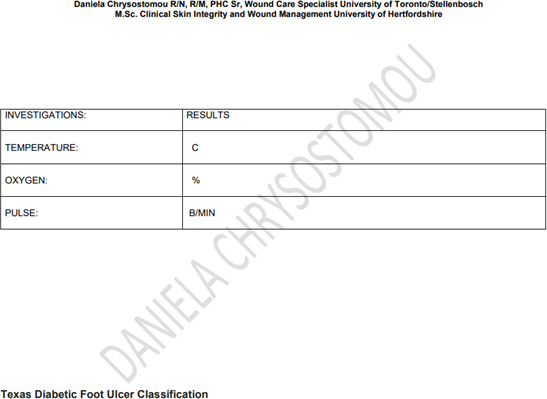


**Figure:**