# Supplementary figures and images for: Medical-Grade Honey Is a Versatile Wound Care Product for the Elderly
Source: JAR Life. 2024 May 17;13:51–9. doi: 10.14283/jarlife.2024.7 (PMC11106090; doi:10.14283/jarlife.2024.7)

**Appendix 1**


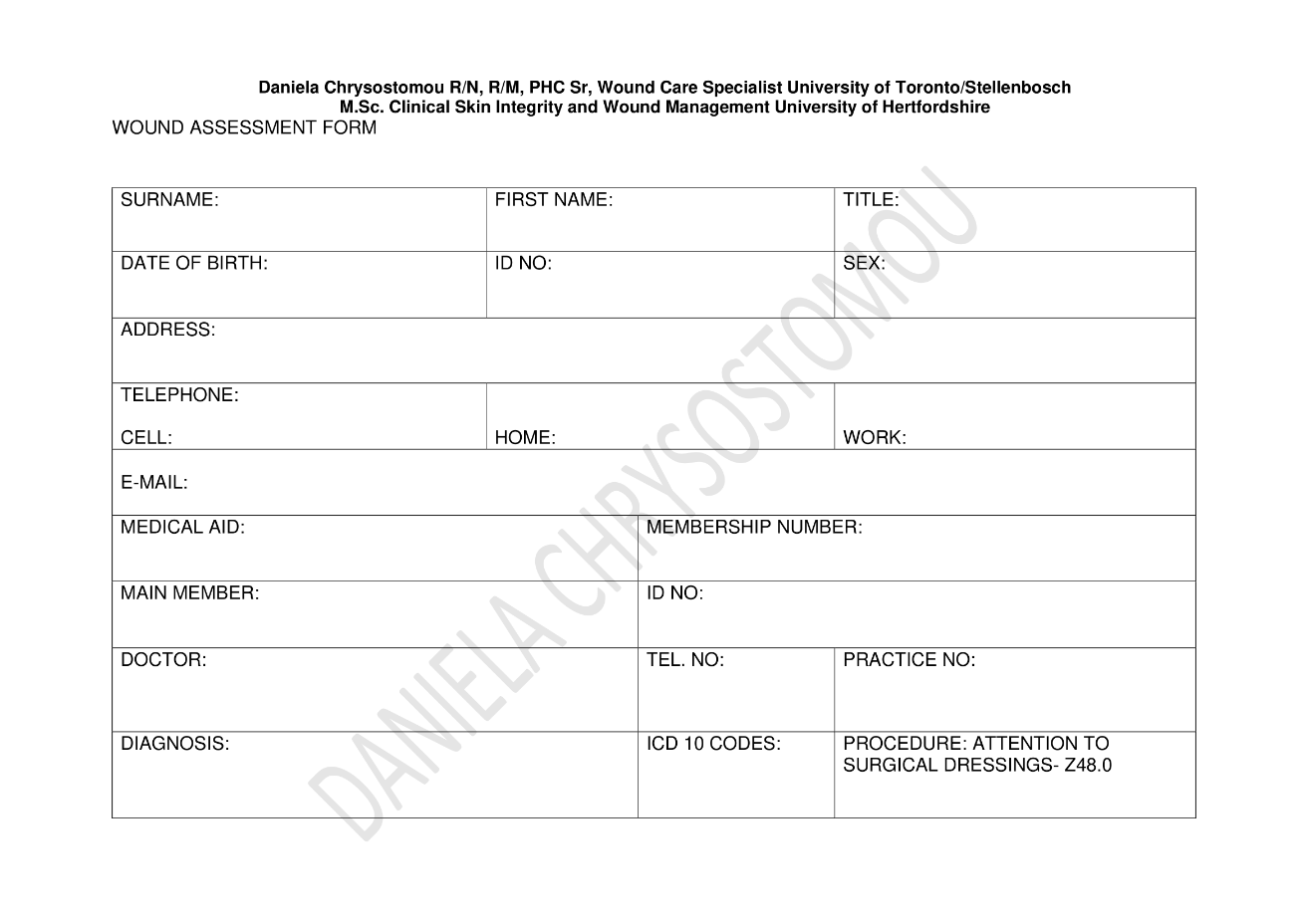

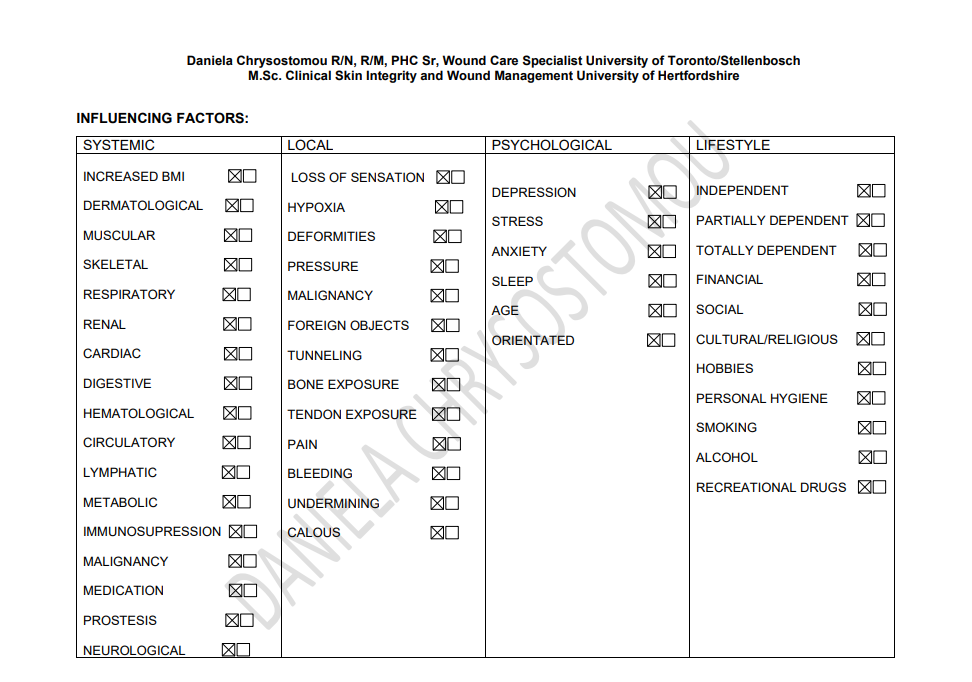

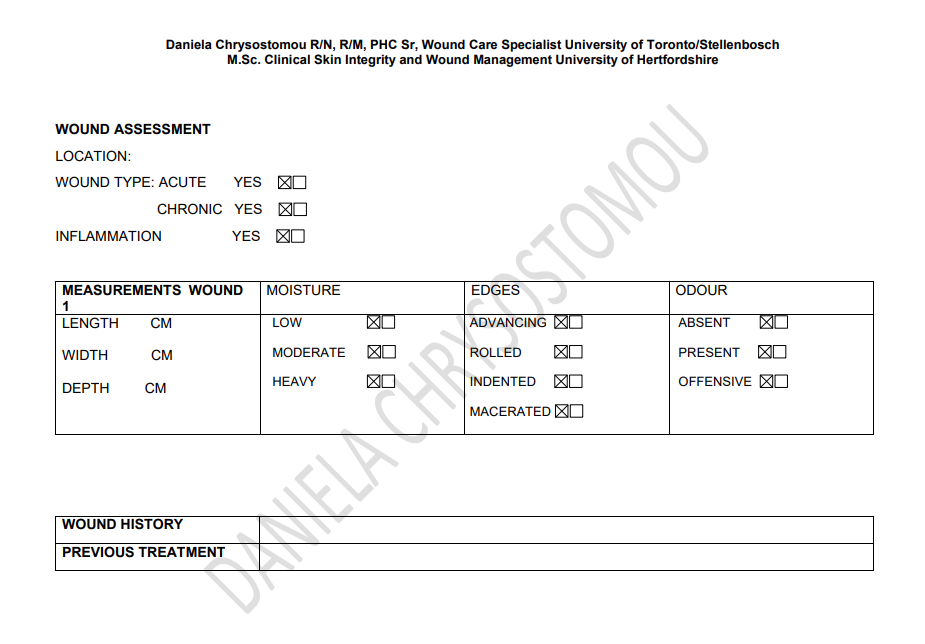

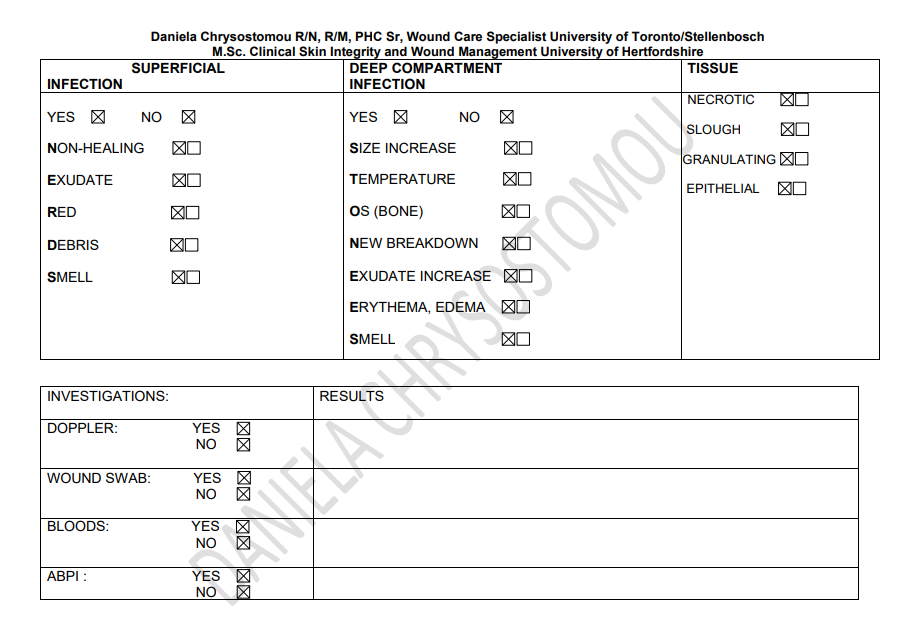

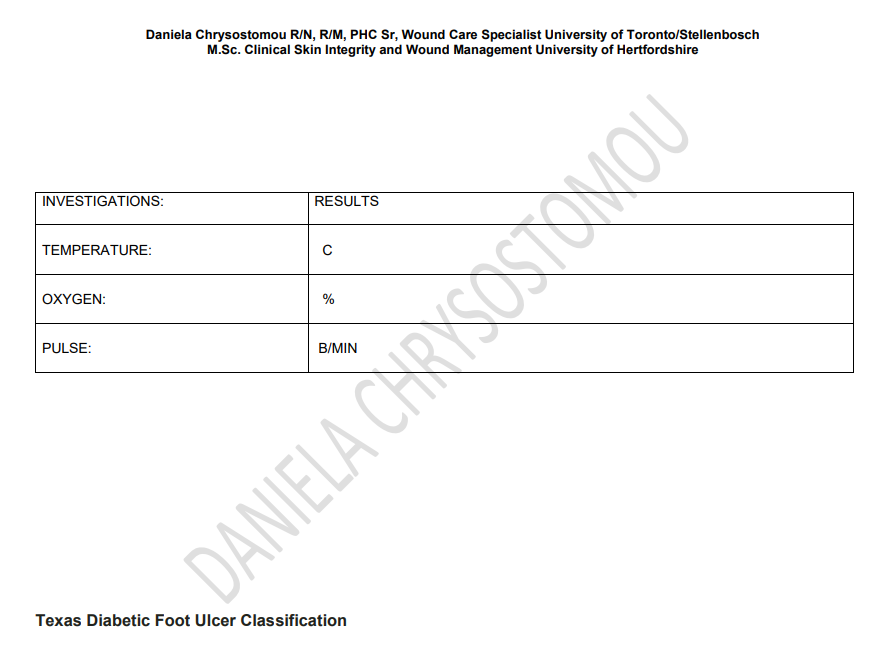

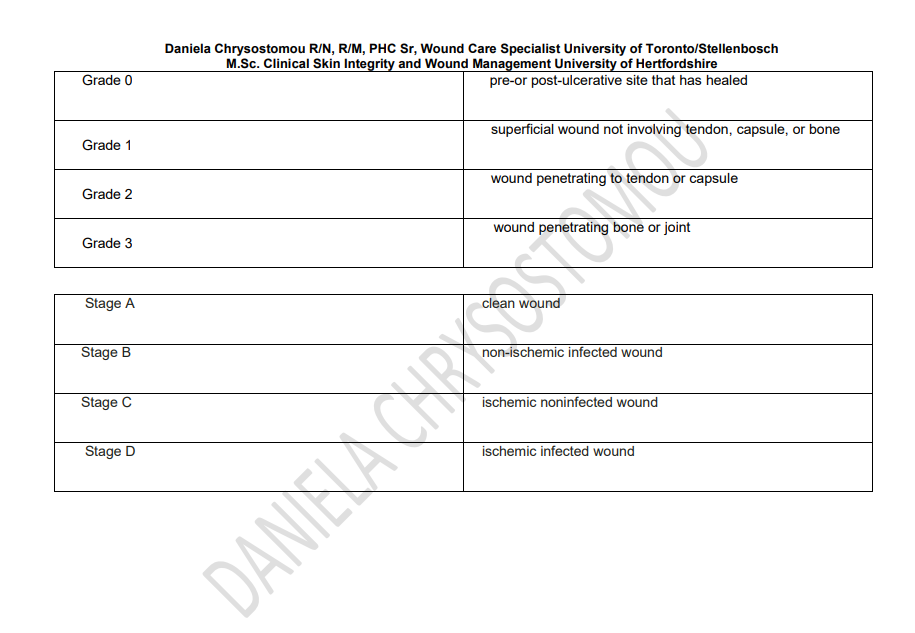

Supplement: Supplementary file 1 [file jarlife-13-0007-S1.docx]
